# Origin of the enhanced photocatalytic activity of (Ni, Se, and B) mono- and co-doped anatase TiO_2_ materials under visible light: a hybrid DFT study†

**DOI:** 10.1039/d0ra07781j

**Published:** 2020-11-26

**Authors:** Hanan H. Ibrahim, Adel A. Mohamed, Ismail A. M. Ibrahim

**Affiliations:** Department of Chemistry, Faculty of Science, Helwan University 11795 Cairo Egypt ismail.ibrahim@science.helwan.edu.eg

## Abstract

The characteristic properties of TiO_2_ (anatase) make doping necessary to enhance its photocatalytic activity. Herein, a density functional theory (DFT) study using the Heyd–Scuseria–Ernzerhof (HSE) hybrid functional was performed to precisely investigate the effect of mono- and co-doping (Ni, Se and B) on the structural, electronic and optical properties of anatase TiO_2_. Notably, the origin of the enhanced photocatalytic activity of the modified systems was determined. The response to visible light was enhanced for all the mono- and co-doped materials except for B_int_, and the highest absorption coefficient was observed for Se^4+^ mono-doping and Se/B_int+sub_ and Ni/B_sub_ co-doping. The decrease in bandgap is associated with a red shift in the absorption edges with the smallest bandgap calculated for Ni/B_sub_ (2.49 eV). Additionally, the Ni, Se^4+^ and Se^2−^ mono-doped systems and Ni/Se^4+^ co-doped systems are proposed as promising photocatalysts for water splitting applications and further experimental validation. Moreover, the Ni/B_int+sub_ and Se/B_int+sub_ co-doped materials can also be valuable photocatalysts for other energy applications due to their enhanced visible light activity and the prolonged lifetime of their produced charge carriers.

## Abstract 2

Hybrid DFT calculations demonstrate that Ni, Se^4+^ and Se^2−^ mono-doped and Ni/Se^4+^ co-doped TiO_2_ are potential photocatalysts for water splitting and hydrogen production.

## Graphical abstract

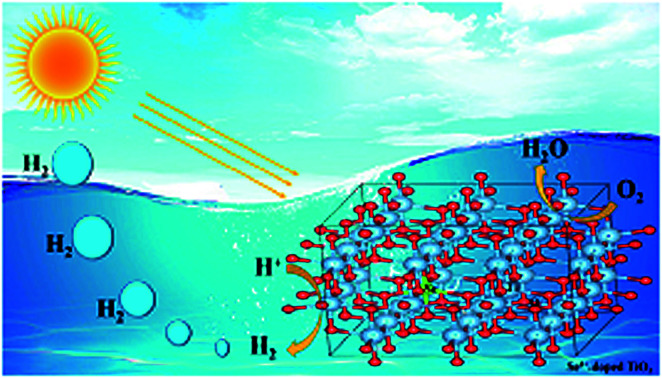

## 1. Introduction

In recent years, clean energy technology has attracted great interest for a sustainable economy and reduction of environmental pollution.^1,2^ Accordingly, photocatalytic materials have been extensively investigated, especially semiconductor photocatalysts. The light absorbed by a semiconductor is characterized by its electronic bandgap. Therefore, TiO_2_ is an ideal photocatalyst due to its characteristic properties such as low cost, low toxicity, and high thermal stability. However, due to the wide bandgap (3.2 and 3.00 eV for anatase and rutile, respectively) of pure TiO_2_, the absorption of light is limited to only UV light, which represents ∼5% of solar energy. Therefore, a low quantum yield of the photo-generated oxidative species is obtained. Thus, bandgap engineering of TiO_2_-based materials is of major interest for solar energy applications such as water splitting for H_2_ production.^3–6^ Doping with ions such as transition metals, rare earth metals, alkali metals, noble metals and non-metals was found to be an effective method for controlling the electronic structure and extending the light response to the visible light region.^7^ Various experimental and theoretical studies have been carried out on TiO_2_ materials, but nearly all the theoretical investigations were demonstrated based on *ab initio* DFT calculations.^8–11^

Nickel doping was largely exhibited since the ionic radius of Ni^2+^ ions is slightly higher than that of Ti^4+^ ions.^9^ Several experimental methods have been employed for the synthesis of Ni-doped TiO_2_ such as the electrospinning technique,^12^ co-precipitation method,^13^ hydrothermal method,^14^ and sol–gel method.^15^ The visible light response and enhanced photocatalytic performance of Ni-doped anatase have been extensively reported.^14,16^ Based on diffuse reflectance spectroscopy (DRS) analysis, bandgap narrowing increases as a function of the dopant concentration.^14,16^ Quantum mechanical methods based on density functional theory were used to explain the optical response and changes in the electronic properties of TiO_2_ upon Ni doping.^17^ On the other hand, several reports on selenium doping were recently published, suggesting that it exhibits several oxidation states, including Se^4+^, Se^0^ and Se^2−^.^18^ Although, cationic Se^4+^ and Se^0^ have been significantly assigned experimentally, Se^2−^ was only clarified theoretically in the previous work by Harb.^10^ Xie *et al.* attributed the narrowed gap (2.19 eV) and the suppressed electron/hole recombination rate upon Se^4+^ doping to the energy states appearing in the gap region upon the incorporation of Se.^11^ A DFT-based study with HSE level showed the effect of Se doping at different valence states with different concentrations on water splitting.^10^ The results showed that substitutional cationic and anionic Se can induce the best band positions for the water splitting reaction. Nonetheless, substitutional Se^2−^ within the same crystal provides localized gap states capable of limiting the hole mobility and enlarging the recombination rate.^10^

For boron-doped TiO_2_, it was found that B doping has photochemical^19,20^ and electrochemical applications,^21^ such as degradation of atrazine as an organic pollutant on light absorption and high performance anode for sodium ion batteries. Based on previous experimental and theoretical studies, B can occupy different sites in the crystal lattice of anatase TiO_2_, either interstitial or substitutional sites, and at high concentration it can occupy both sites.^20^ For interstitial B, no visible light activity was observed; however, a response to visible light was detected for substitutional B due to the electronic transitions from the deep states.^22,23^ At high concentration, both interstitial and substitutional B were found to be stable in bulk anatase or co-doped with other elements.^20,23^ B was co-doped with Ni by Masae *et al.*, who claimed the improvement of photocatalytic activity upon co-doping, but the origin of this enhancement was not clear.^24^

To the best of our knowledge, neither Ni/Se co-doping nor Se/B co-doping has been addressed experimentally and theoretically to date. Therefore, this study proposes new co-doped TiO_2_ photocatalysts for further experimental validation. In the current work, we attempted to study the effect of mono-doping and co-doping with different metals and non-metals at the HSE hybrid functional level on the photocatalytic activity of anatase TiO_2_. In our calculations, the structural, electronic, and optical properties were addressed and compared to the available experimental data. In addition, a systematic description of the induced levels on defect formation and the interaction between these states on co-doping were exhibited. Besides, the formation energy was also calculated, revealing the stability upon the introduction of the dopant to the anatase lattice. We found that most dopants reduced the bandgap, with a significant improvement for co-doped materials. The visible light response and the value of the absorption edge were determined from UV-Vis absorption spectra calculated at the HSE hybrid functional level together with the frequency-dependent dielectric function. The photocatalytic reaction of TiO_2_-based materials was investigated by determining the band edge position of modified materials with respect to the normal hydrogen electrode (NHE) potential for water splitting.

## 2. Computational details

In our work, spin-polarized DFT calculations were performed on pure and defected anatase using projector augmented plane wave methods for treating the valence electrons and their corresponding ionic cores^25^ as implemented in the VASP code.^26,27^ A grid cutoff energy of 560 eV and 2 × 2 × 2 Monkhorst–Pack *k*-point meshes were converged for the plane wave basis set and for sampling the first Brillouin zone, respectively.^28^ Gaussian smearing with a value of 0.1 eV was used for the Brillouin zone integrations during the total energy calculations. The doped systems were constructed from 3 × 3 × 1 anatase supercells corresponding to 108 atoms. The valence electron configurations in the PAW potentials of the considered elements were Ni 3d^8^ 4s^2^, Se 4s^2^ 4p^4^, B 2s^2^ 2p^1^, Ti 3d^3^ 4s^1^ and O 2s^2^ 2p^4^. For the geometry optimization and formation energy calculations, the generalized gradient approximation (GGA) together with the Perdew, Burke, and Ernzerhof (PBE)^29^ exchange-correlation functional was employed. However, the GGA functional fails to produce accurate results, especially when addressing band characteristics such as bandgap values for the strongly correlated d and f electrons. Thus, to overcome this deficiency, a hybrid functional was used by introducing the Hartree–Fock exchange in the DFT calculations such as the screened hybrid HSE06 functional, which was applied to reproduce more effective band gap values comparable to the experimental values. Therefore, the Heyd–Scuseria–Ernzerhof (HSE) hybrid functional was used to evaluate the crystal structure and electronic properties.^30–32^ The screening parameter of 0.207 Å^−1^ was used as applied in the standard HSE06 functional.^32^ However, the exact exchange energy was optimized by fitting the calculated band gap to the experimental value of pure anatase by checking several values (25%, 22%, 20%, 18%, 17% and 15%), as performed in our recent work.^33^ Hereinafter, we refer to this functional as the HSE hybrid functional.

## 3. Results and discussion

### 3.1. Dopant effect on the structural and electronic properties

**Fig. 1:**
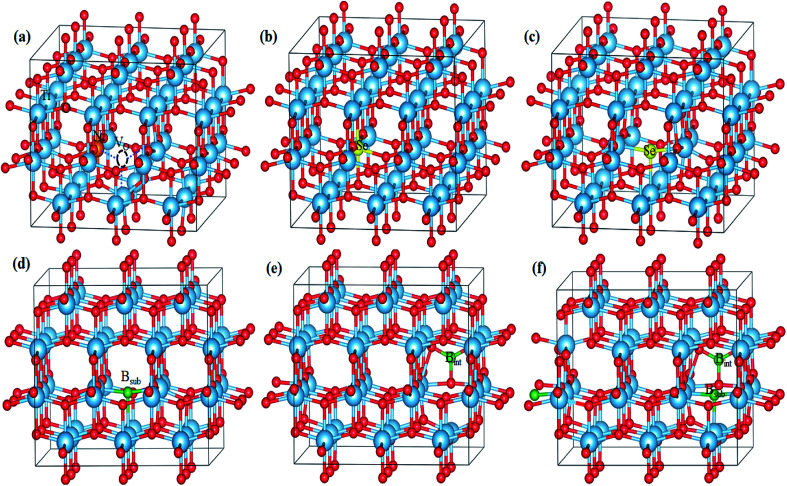
Optimized geometries of (a) Ni-doped, (b) Se^4+^-doped, (c) Se^2−^-doped, (d) B_sub_-doped, (e) B_int_-doped, and (f) B_int+sub_-doped anatase TiO_2_ determined using the HSE hybrid functional level.

**Fig. 2:**
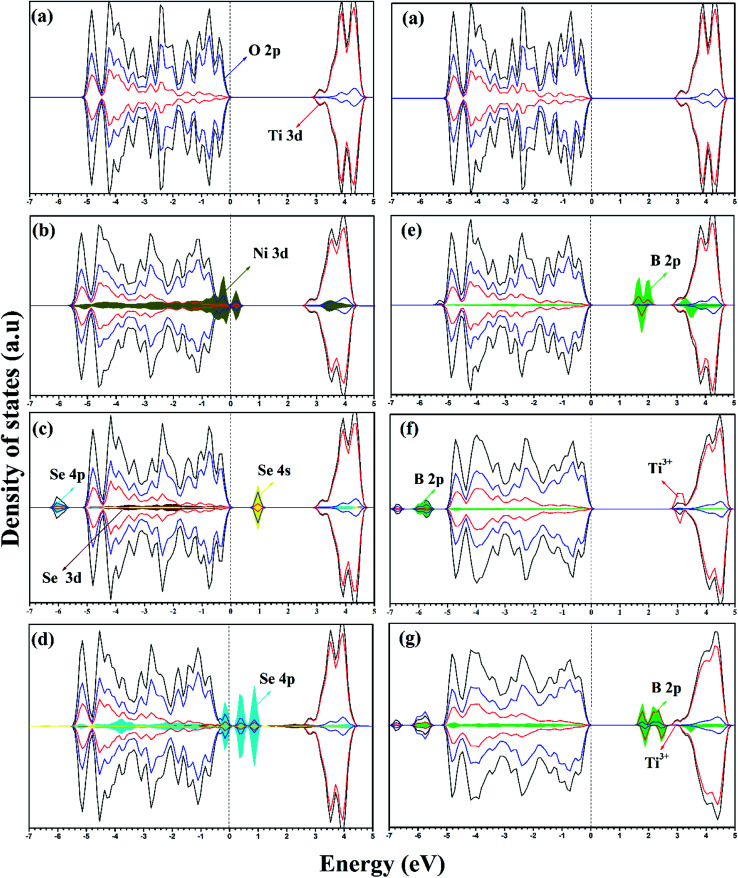
DOS and PDOS of (a) pure anatase TiO_2_, (b) Ni-doped, (c) Se^4+^-doped, (d) Se^2−^-doped, (e) B_sub_-doped, (f) B_int_-doped, and (g) B_int+sub_-doped anatase TiO_2_ calculated using the HSE hybrid functional level. The valence band maximum is set at 0 eV.

For pure anatase TiO_2_, the calculated lattice parameters are *a* = 3.834 Å and *c* = 9.624 Å, which agree with the experimental lattice parameters of *a* = 3.784 Å and *c* = 9.502 Å.^34^ Upon the introduction of impurity into the anatase lattice, volume distortions were observed since the doped impurity has a different configuration and ionic radius from the replaced ions of the host lattice. The lattice parameters and cell volumes of the modified systems are listed in Table 1, and the relaxed structures corresponding to the mono-doped materials are shown in Fig. 1. For all the systems, the lattice parameters of the relaxed structures increased due to the incorporation of an impurity. The bandgap value of pure anatase calculated with the HSE hybrid functional was about 3.26 eV, which is consistent with the experimental value (3.2 eV) and the previous HSE hybrid functional calculations.^35,36^ The DOS and PDOS calculations for pure, mono-doped, and co-doped anatase TiO_2_ are shown in Fig. 2. The analysis of PDOS for pure anatase shows hybridization between the O 2p and Ti 3d states, indicating the covalent character of the formed Ti–O bond, as shown in Fig. 2a.^33^ In our work, the bandgap represents the energy difference between the valence band maximum (VBM) to the conduction band minimum (CBM). In contrast, the energy gap signifies the energy difference between the highest occupied state and the lowest unoccupied state. The electronic structure also changes upon TiO_2_ doping since new states appear in the bandgap region. The position of these gap states may affect the visible light response and the charge separation ability of modified TiO_2_. If these states are shallow, they act as trapping centers for charge carrier pairs, endowing TiO_2_ with longer life photogenerated charges. However, if the impurity states move high in the bandgap, they are deep states and can act as recombination centers for the electron/hole pairs, suppressing the photoactivity.^37,38^

**Table 1:** The optimized and the experimental (in brackets)^18,34^ lattice parameters, cell volume and the volume difference (Δ*V*) upon the formation of defects for pure and modified anatase TiO_2_ calculated based on the HSE hybrid functional level

System	Lattice parameter		Cell volume (Å^3^)	Δ*V* (Å^3^)
	*a* (Å)	*c* (Å)		
Pure TiO_2_	3.835 (3.784)	9.624 (9.502)	1273.91	—
Ni–TiO_2_	3.838 (3.789)	9.631 (9.715)	1276.78	2.78
Se^4+^–TiO_2_	3.839 (3.760)	9.633 (9.490)	1277.62	3.71
Se^2−^–TiO_2_	3.851	9.665	1290.13	16.22
B_sub_–TiO_2_	3.842	9.642	1280.83	6.92
B_int_–TiO_2_	3.846	9.652	1285.14	11.23
B_int+sub_–TiO_2_	3.851	9.664	1289.90	15.99
Ni/B–TiO_2_	3.840	9.637	1278.91	5.00
Ni/B_int+sub_–TiO_2_	3.851	9.665	1290.33	16.42
Se^4+^/B–TiO_2_	3.849	9.659	1287.71	13.80
Se^4+^/B_int+sub_–TiO_2_	3.864	9.699	1303.85	29.94
Ni/Se^4+^–TiO_2_	3.838	9.631	1276.69	2.78
Ni/Se^2−^–TiO_2_	3.851	9.663	1289.55	15.64

In the case of the mono-doped materials, the replacement of Ti^4+^ by Ni^2+^ leads to the formation of oxygen vacancy to preserve the charge neutrality, showing a stoichiometry of Ni_*x*_Ti_1−*x*_O_2−*δ*_ where (*x* = 0.028 and *δ* = 0.028), and the relaxed geometry is shown in Fig. 1a. The calculated Ni–O bond length is 2.06 Å, which is slightly longer than the reference Ti–O bond due to the ionic radii differences. The calculated PDOS of the Ni mono-doped anatase (Fig. 2b) shows hybridization between the Ni 3d and O 2p states, which confirms the existence of an Ni–O–Ti bond. The incorporation of Ni into the anatase TiO_2_ lattice preserves the diamagnetism of the system with a singlet spin state and a magnetic moment of 0 *μ*_B_.^39^ Moreover, it can be observed that the Ni states are completely overlapped with the VBM, with some states located at 2.53 eV from the CBM. Therefore, the main bandgap is reduced to 2.89 eV with an energy gap of 2.53 eV, which is consistent with the previous results by Blanco-Vega *et al.*^15^ for Ni doped at small concentrations. Thus, based on the bandgap reduction, enhanced photoactivity towards visible light can be observed upon Ni mono-doping.

For Se-modified anatase, substitutional Se is doped at two different sites with two different stoichiometries.^18^ In one structure, Se^4+^ is substituted at the Ti^4+^ site with the formula of Se_*x*_Ti_1−*x*_O_2_, whereas Se^2−^ at the oxygen site leads normally to TiO_2−*δ*_Se_*δ*_. For the configuration with cationic Se, the obtained Se–O bond has a length of 2.00 Å, whereas the Ti–Se bond formed upon anionic Se doping of 2.30 Å is much longer. For both cases, a closed-shell singlet spin state was observed, corresponding to the diamagnetic properties of the formed structures. The DOS and PDOS for the cationic Se depicted in Fig. 2c shows a slight increase (0.03 eV) in the main bandgap value on the formation of the Se^4+^ defect; however, the energy gap originating from the incorporation of Se states in the bandgap region is about 2.18 eV. The energy gap calculated for Se^4+^ mono-doping is consistent with the previously observed value.^11,40^ The slight increase in the calculated main band gap was reported using the standard HSE06 functional.^10^ The calculated main bandgap value for two Se^4+^ doped anatase within the standard HSE06 functional was about 3.4 eV, whereas the calculated bandgap value of pure anatase was 3.3 eV.^10^ However, as shown in Table 2, Se^4+^ doping decreases the band gap of TiO_2_ experimentally.^18^ This can be attributed to the anatase to rutile phase transition on Se doping, where the anatase percentage was 50% at the calcination temperature of 650 °C.^18^ The deep states formed in the bandgap region is mainly due to the Se 4s states with a small contribution from the Se 4p states to the valence band (VB).^41^ However, the Se 4p contribution to the conduction band (CB) is noticeable. Furthermore, the Se 4s states are hybridized with the O 2p states, which gives an indication of the strength of the formed Se–O bond. The doped Se impacts the diamagnetic properties of the modified TiO_2_, which is associated with the closed shell singlet state, resulting in a 0 *μ*_B_ net magnetic moment. On the other hand, the anionic Se^2−^-modified anatase structure formed through oxygen substitution reveals different DOS and PDOS from the cationic doped material (Fig. 2d). The main bandgap is reduced to 3.18 eV, while the energy gap is narrowed to 1.88 eV by the Se^2−^ impurity states in the forbidden region.^10^ The gap states are mainly due to the Se 4p states, which are separated by 2.34 and 1.88 eV from the CBM.^41^ Some of these states are overlapped with the VBM, while the other states are hybridized with the O 2p states. For both Se mono-doped cases, the electron transition is allowed from the induced bandgap states to the CB. Consequently, the photocatalytic activity of these materials under visible light is enhanced. Nevertheless, the charge separation efficiency may be affected by these deep states, and consequently the photocatalytic activity. Accordingly, it is worthwhile to co-dope these materials with another elements capable of improving the charge separation efficiency.

**Table 2:** The calculated bandgaps and energy gaps using the HSE hybrid functional level of pure and defected anatase TiO_2_ materials in comparison to the available experimental values^15,18,36^

System	Bandgap (eV)	Exp. bandgap	Energy gap (eV)	Formation energy (*E*_f_)	
				Ti-rich	O-rich
Pure TiO_2_	3.26	3.20	3.26	—	—
Ni–TiO_2_	2.89	2.72	2.53	4.579	0.118
Se^4+^–TiO_2_	3.29	3.05	2.18	7.911	−1.011
Se^2−^–TiO_2_	3.18		1.88	1.155	5.616
B_sub_–TiO_2_	3.21		1.04	3.873	8.334
B_int_–TiO_2_	3.52		0.23	0.635	0.635
B_int+sub_–TiO_2_	3.40		0.23	−3.568	7.276
Ni/B_sub_–TiO_2_	2.49		1.10	5.921	5.291
Ni/B_int+sub_–TiO_2_	2.78		0.31	5.331	5.330
Se^4+^/B_sub_–TiO_2_	2.98		1.23	10.088	5.627
Se^4+^/B_int+sub_–TiO_2_	3.31		0.26	8.512	4.050
Ni/Se^4+^–TiO_2_	2.87		2.50	11.680	−1.703
Ni/Se^2−^–TiO_2_	3.11		1.55	3.893	3.893

The B-doped TiO_2_ was simulated by replacing B^2−^ at the O^2−^ site with the formula of TiO_2−*δ*_B_*δ*_, and inserting B into the anatase lattice, leading to TiO_2_B_*δ*_. The mixed interstitial-substitutional B system was also modeled through substitution of O^2−^ by B from the anatase supercell with the incorporation of an additional B into the crystal system as TiO_2−*δ*_B_2*δ*_. In the anatase lattice, B_sub_ is bound to three neighboring Ti ions with three bonds, *i.e.* two short (2.12 Å) and one long (2.39 Å) bonds (Fig. 1d).^22^ Boron added to the lattice has an odd number of valence electrons, indicating the paramagnetic properties of the neutral defect. In our study, we considered the doublet spin state, and thus the third electron is shared with a single Ti rather than bonding, explaining the longer bond length. The electrons occupy the hybrid B 2p–Ti 3d states localized deep in the bandgap region at about 1.34 and 1.04 eV for the spin up states and 1.22 eV for the spin down state below the conduction band minimum (CBM). The electronic transitions from these states can occur on visible light absorption; however, these deep states can suppress the carrier separation. Moreover, the main bandgap is slightly red shifted from the anatase bandgap, in agreement with the previous experimental observations.^38,42^ For interstitial B (B_int_), it was reported that B_int_ may be tricoordinate [BO_3_] or tetracoordinate [BO_4_].^22^ However, the electronic characteristics of interstitial boron were rather independent of the site where the boron atom is incorporated.^22^ Therefore, we only considered a [BO_3_] structure in our work, as shown in Fig. 1e. The interstitial B atom is bonded to the three vicinal oxygen ions, where two bonds are similar (1.4 Å) and the third is shorter (1.376 Å). In this case, the boron exhibits a trigonal planer coordination.^26^ According to the PDOS, as shown in Fig. 2f, it is observed that no states due to boron contribution are formed in the bandgap, while the B 2p states are located below the valence band.^22^ The states incorporated and overlapped with the CBM are mainly due to the Ti^3+^ ions, which can trap the photo-generated carriers and enlarge the lifetime of these carries.^43^ This result indicates that the B electrons are partially delocalized and consequently donated to the lattice forming B^3+^.^22^ Despite the Ti^3+^ states, the main bandgap is blue shifted upon the introduction of interstitial boron, and no visible light response is detected for that system.^23,44^ The blue-shifted bandgap is attributed to the existence of BO_*x*_, which has a high bandgap compared to pure anatase.^22,45^ The situation for B_int+sub_ is different since the concentration of B in the lattice is increased by the introduction of two B atoms, *i.e.* one at an interstitial position and the other at the substitutional position, replacing an oxygen ion from the lattice, as shown in Fig. 1f. With this coupling, the bond lengths change from the mono-doped case. The three bonds surrounding the interstitial B are equal (1.379 Å), whereas the bonds around the substitutional B are still two short and one long bond (2.07 and 2.12 Å), respectively. The PDOS of B_int+sub_ (Fig. 2g) shows that the gap states move high in the bandgap towards the CB. Moreover, two of the shallow levels seen in B_int_ almost disappear in B_int+sub_ by the interaction between B_int_ and B_sub_ with overlap between their states. However, one shallow state due to Ti^3+^ is still observed below the CB, which may be associated with enhanced electron trapping. The main bandgap is slightly red shifted from B_int_, but still higher than the anatase bandgap.

**Fig. 3:**
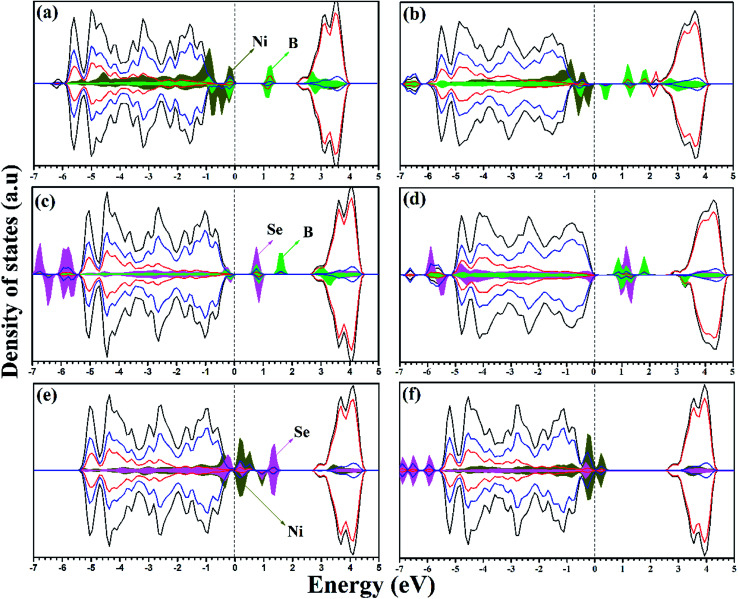
DOS and PDOS of (a) Ni/B_sub_ co-doped, (b) Ni/B_int+sub_ co-doped, (c) Se^4+^/B_sub_ co-doped, (d) Se^4+^/B_int+sub_ co-doped, (e) Ni/Se^2−^ co-doped, and (f) Ni/Se^4+^ co-doped anatase TiO_2_ calculated using the HSE hybrid functional level. The valence band maximum is set at 0 eV.

On co-doping, the Ni/B_sub_, Ni/B_int+sub_, Se^4+^/B_sub_, Se^4+^/B_int+sub_, Ni/Se^2−^, and Ni/Se^4+^ co-doped systems are associated with Ni_*x*_Ti_1−*x*_O_2−2*δ*_B_*δ*_, Ni_*x*_Ti_1−*x*_O_2−2*δ*_B_2*δ*_, Se_*x*_Ti_1−*x*_O_2−*δ*_B_*δ*_, Se_*x*_Ti_1−*x*_O_2−*δ*_B_2*δ*_, Ni_*x*_Ti_1−*x*_O_2−2*δ*_Se_*δ*_, and Ni_*x*_Se_*x*_Ti_1−2*x*_O_2−*δ*_, respectively. For all the co-doped materials, *x* = 0.028 and *δ* = 0.028 if they exist. For the co-doping models, different configurations were considered by distributing the dopants (adjacent and separated) in the supercell. It was found that the formation of adjacent dopants is more energetically favorable with respect to other configurations.^46^ Therefore, the lowest energy structures were selected to examine their electronic and optical properties and their catalytic activity (see ESI† for fractional coordinates). Ni/B_sub_ co-doping exhibits different ionic features from the mono-doped materials. The PDOS for Ni/B_sub_ shows an interaction between the two dopants, and overlapping is observed between the Ni 3d and B 2p states due to the bond formed between Ni and B. Also, the Ni 3d states are completely overlapped with the VBM, giving rise to bandgap narrowing of (2.49 eV); however, the B states appear deep in the bandgap region and separated by 1.10 eV from the CBM. Upon geometrical optimization of the Ni/B_Sub_ co-doped material, the local magnetic moment over the Ni ion becomes 0.843 *μ*_B_; however, the magnetic moment over the whole lattice is 1.02 *μ*_B_. In the Ni/B_int+sub_ co-doped system, an interaction exists between Ni and boron at the VBM and the total magnetic moment is 1.886 *μ*_B_, while the local magnetic moment on Ni ion is 1.182 *μ*_B_. Herein, the Ni 3d states are still overlapped with the VBM; however, most of the B states are found deep in the bandgap (Fig. 3b). Moreover, there are states with shallow character, corresponding to the formation of Ti^3+^. Due to these impurity states, many transitions can be observed upon visible light illumination and the charge carrier separation can be enhanced by the existing shallow states, in addition to the red shift in the calculated main bandgap (2.78 eV).

In the Se/B_sub_ co-doped anatase lattice, the PDOS and DOS is illustrated in Fig. 3c. The Se-occupied states can be observed at about 0.91 eV above the VBM, which overlap with the B states due to Se–B bond formation, while a separated B state is introduced in the bandgap at about 1.23 eV from CBM, indicating the allowed electron transitions on visible light irradiation. Moreover, the band gap is narrowed to 2.98 eV with a total magnetic moment of 0.67 *μ*_B_ for the whole system. The deep B states provide centers from which electrons can be excited on light absorption. Therefore, the charge carrier population increases; however, they may act as recombination centers for these carriers. Thus, the photocatalytic activity of Se^4+^/B is enhanced by the response to visible light, although the lifetime of the photo-produced carriers may be affected. Se/B_int+sub_ displays different electronic structures, and its calculated DOS and PDOS are illustrated in Fig. 3d. Similar to Ni/B_int+sub_, the interaction between Se and B increases upon the incorporation of interstitial B. Moreover, the deep states due to substitutional B observed on Se/B_sub_ co-doping are placed in the forbidden gap at about 1.41 eV from the CBM on Se/B_int+sub_ co-doping. In addition, a shallow Ti^3+^ state appears, which is overlapped with the CBM. The calculated DOS analysis indicates that the bandgap is slightly blue shifted (3.31 eV); however, the visible light response can be enhanced by the transitions from the previously mentioned bandgap states. In addition, the shallow state can prolong the lifetime of the carriers. Thus, Se/B_int+sub_-modified anatase TiO_2_ can be suggested as an enhanced photocatalyst.

Upon Ni/Se^2−^ co-doping, the calculated bandgap is about 3.11 eV with the impurity states overlapping with the VBM in addition to the bandgap states separated by about 1.55 eV from the CBM. The PDOS with this co-doping shows a higher contribution of Ni 3d and Se states to the VB, as shown in Fig. 3e. According to the DOS calculations, it can be confirmed that the Se states are overlapped with the top of the VB, and the deep states exited in the bandgap are mainly due to the Se 4p states. Therefore, the Se^2−^, states which are either overlapped with Ni or isolated at about 1.56 eV from the VBM in Ni/Se^2−^, are due to the contribution of the Se 4p states. In the second case, Ni/Se^4+^ is co-doped as dual metal doping by the substitution of two close Ti ions. According to the DOS and PDOS shown in Fig. 3f, a state is observed at 2.50 eV from the CBM, indicating that more carriers are generated on visible light absorption. The calculated band gap is about 2.87 eV, which is higher than the case of Ni/Se^2−^ co-doping, but still lower than pure TiO_2_, revealing a better response to visible light. For both materials (Ni/Se^4+^ and Ni/Se^2−^), the photocatalytic activity under visible light is improved due to the bandgap reduction and higher number of photogenerated carriers, but the deep states may affect the stability of the photogenerated carriers.

### 3.2. Formation energy calculations

To reveal the stability and possibility of defect doping in the anatase lattice, the defect formation energy (*E*_f_) of the modified systems was calculated using the following equation:

1 *E*_f_ = *E*_doped_ − *E*_undoped_ − (*n*_M_*μ*_M_ + *n*_N−M_*μ*_N−M_) + (*n*_O_*μ*_O_ + *n*_Ti_*μ*_Ti_)

where *E*_doped_ and *E*_undoped_ are the calculated total energy of the pure and defected anatase TiO_2_, and *n* represents the number of added or removed dopant and host ions. *μ*_M_ and *μ*_N−M_ are the total energy per atom of metal and non-metal dopants calculated from their bulk, respectively. The formation energy of TiO_2_ based photocatalysts is dependent on the growth conditions and vary under Ti-rich and O-rich conditions. For pure TiO_2_, *μ*_O_ and *μ*_Ti_ should satisfy the thermodynamic relationship (*μ*_TiO_2__ = *μ*_Ti_ + 2*μ*_O_).^37^ The relationship between the *μ*_O_ and *μ*_Ti_ chemical potentials and the formation energy was explained in our recent work.^33^ The formation energy values for each doped material under both conditions are shown in Table 2.

The lower the formation energy of defects, the more likely they are formed. Under O-rich conditions, the formation energy of metal mono-doping is lower than that of non-metal mono-doping. On the other hand, Se^2−^ or B doping shows higher stability under Ti-rich conditions. Under both conditions, the formation energy is higher for B_sub_ than B_int_ and B_int+sub_, which can verify the previously mentioned discussion of the metastable character of substitutional boron.^22,33^ The B_int+sub_ defect formation energy is the lowest value (−3.568 eV), suggesting that B at high concentration tends to occupy both the interstitial and substitutional sites.

The calculated formation energy for the co-doped defects demonstrates that defect co-doping is more facilitated under O-rich than Ti-rich conditions. However, the values for all the systems are more positive than the mono-doped cases except for Ni/Se^4+^, which exhibits a higher negative formation energy than Ni or Se^4+^ mono-doping. Moreover, Ni/B_sub_, Ni/B_int+sub_ and Ni/Se^2−^ co-doping and B_int_ mono-doping show the same value of formation energy under both conditions, indicating the independence of formation from the growth conditions.

### 3.3. Dopant effect on the optical properties

The linear optical properties can be obtained depending on the frequency-dependent dielectric function: *ε*(*ω*) = *ε*_1_(*ω*) + i*ε*_2_(*ω*), where *ε*_1_and *ε*_2_ represent the real and imaginary parts of the dielectric function, respectively, and *ω* is the photon frequency. The imaginary part *ε*_2_(*ω*) can be calculated from the momentum matrix element between the VB and CB wavefunctions, whereas the real part *ε*_1_(*ω*) can be obtained from the Kramers–Krönig relationship.^47^ The UV-Vis optical absorption can be calculated by determining the optical absorption coefficient *α*(*ω*) for each material (in cm^−1^) as a function of the wavelength of the incident light according to the following equation:^48^2
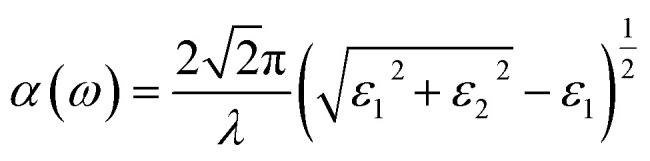
where λ and *ω* represent the wavelength and frequency of incident light radiation, respectively.^49^

**Fig. 4:**
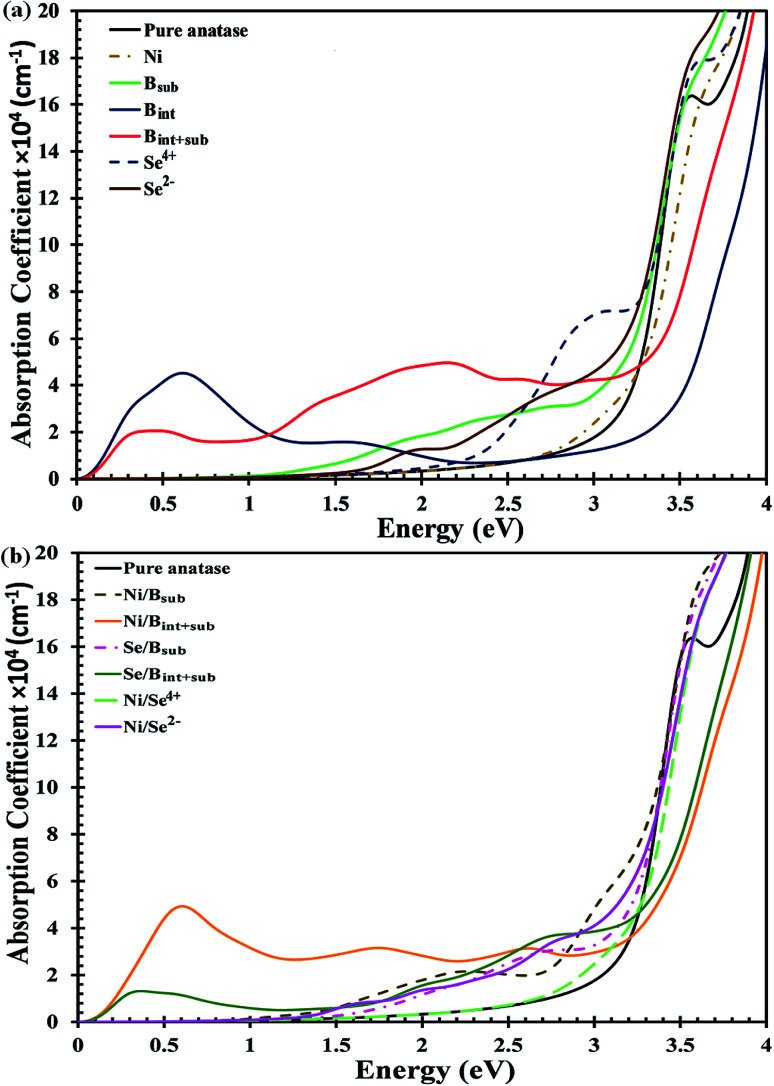
Calculated absorption coefficients with HSE hybrid functional for (a) mono-doped, and (b) co-doped anatase TiO_2_ materials.

The UV-Vis optical absorption spectrum of anatase TiO_2_ is illustrated in Fig. 4a. The calculated bandgap for pristine anatase TiO_2_ is about 3.26 eV, which is about 0.06 eV higher compared to the experimental value.^33^ Therefore, all the anatase TiO_2_-based applications are limited to UV light, which contributes about ∼5% to solar irradiation. The spectra of the mono-doped and co-doped materials over pure anatase TiO_2_ are depicted in Fig. 4a and b, respectively. For all the materials, the absorption edge is red shifted considering the reference spectrum of pure TiO_2_ except for the B_int_-doped materials. For the Ni mono-doped system, the light response extends up to about 2.5 eV, whereas the absorption edges are red shifted to about 1.6 and 2.2 eV for Se^2−^ and Se^4+^ mono-doping, respectively. In B mono-doping, three different spectra for the three configurations B_sub_, B_int_ and B_int+sub_ are observed. For B_sub_, the absorption edge is red shifted to about 1.04 eV with a high absorption coefficient in the visible light region besides the absorption due to the main bandgap transition shown at about 3.18 eV. B_int_ and B_int+sub_ show different trends, where the absorption edge for both is blue shifted with absorption coefficients in the IR region, which are consistent with the states observed below the CB in the DOS.

Upon co-doping, all the co-doped materials show an extended visible light response, indicating improved photoactivity over mono-doped materials. Ni/B_int+sub_ co-doping exhibits high absorption efficiency, indicating a high population of photogenerated charge carriers in addition to the red-shifted absorption due to the main bandgap excitations. Moreover, intense peaks are detected in the IR region, which confirm the transition from the shallow states located directly below the CB. The main absorption peak for Ni/B_sub_ co-doping is formed at about 2.5 eV. In addition, several peaks appear in the visible light region of the spectrum, but with a higher absorption coefficient than that of Ni/B_int+sub_. Upon Se/B_sub_ co-doping, several absorptions can be observed in the visible light region with no absorption in the IR region as a result of the transitions from the deep states to the CB. Se/B_int+sub_ co-doping also shows visible light absorption, but with higher absorption coefficients than that of Se/B_sub_ co-doping. Additionally, a high intensity peak appears in the IR region, confirming the shallow state electron excitation expected from the DOS results. For Ni/Se co-doped anatase, the absorption corresponding to the bandgap excitations is red shifted to visible light for both the Ni/Se^4+^ and Ni/Se^2−^ co-doped materials with energy values of about 2.9 and 3.1 eV, respectively. In addition, various peaks are shown for Ni/Se^2−^ as a result of the electron transitions from the forbidden gap states to the CB.

Consequently, the visible light response is enhanced for all the materials except B_int_-modified anatase. However, the absorption efficiency is better for the Se^4+^ mono-doped and Ni/B_sub_ and Se/B_int+sub_ co-doped systems, revealing the strength of these materials as effective photocatalysts under visible light.

### 3.4. Relative band position for water splitting

Thermodynamically, the photocatalytic reaction of adsorbed species is governed by the position of the edges of the valence band (VBE) and conduction band (CBE) of semiconducting photocatalysts. For water splitting and fuel production or the degradation of organic materials, there are standard conditions that the relevant potential of the acceptor species should lie below the CBE of the photocatalyst (more positive), whereas the potential level of the donor species should be located above the VBE (more negative).^50^

**Fig. 5:**
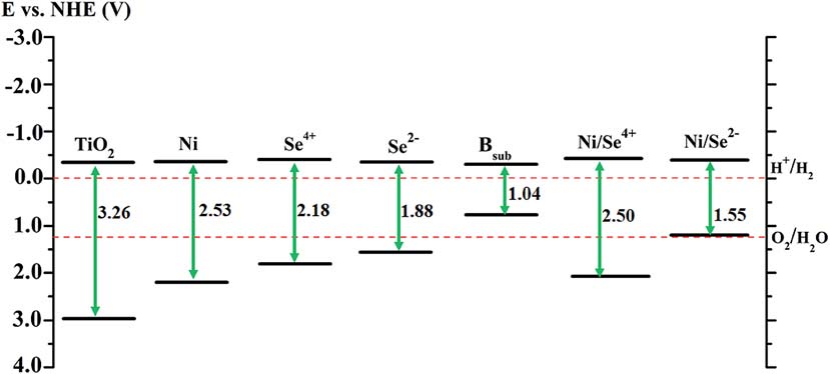
Calculated band positions of mono- and co-doped TiO_2_ anatase materials. The values are with respect to the NHE potential in (V).

Accordingly, the photocatalytic activity of the modified anatase TiO_2_ was evaluated for water splitting as an application for H_2_ production. The values of the VBE and CBE positions of pure anatase were calculated with respect to the normal hydrogen electrode (NHE) potential.^5,6,51^ For the doped system, the positions of edges of the valence and conduction bands were detected from the DOS analysis depending on their relative positions compared to pure anatase TiO_2_.^10^ The calculations for pure anatase TiO_2_ shows that the VBE is located at 2.94 eV, while the CBE position is 0.32 eV more negative than the reduction potential of H^+^/H_2_, which agrees with the previous experimental values.^51^ As shown in Fig. 5, the CBE position of the Se^2−^ mono-doped system is almost similar to that of TiO_2_, whereas the VBE is shifted up by about 1.39 eV over TiO_2_ (about 0.32 eV more positive than the O_2_/H_2_O potential). This reveals that the reduction ability of H^+^ is maintained for Se^2−^ mono-doped TiO_2_ with an increased tendency to release oxygen. In contrast, the CBE positions for the Ni and Se^4+^ mono-doped and Ni/Se^4+^ co-doped systems are shifted up by about 0.02, 0.08, and 0.08 eV over TiO_2_, respectively indicating that the reduction ability of H^+^ remains possible. In addition, their VBE positions are about 0.96, 0.55 and 0.87 eV more positive than the oxidation potential of O_2_/H_2_O, respectively, revealing that the ability to release oxygen is strong. Therefore, all the modified anatase materials, as shown in Fig. 5, can be candidates for water splitting and hydrogen production except for the B_sub_ mono-doped and NiSe^2−^ co-doped systems. In the case of B_sub_ and Ni/Se^2−^, the CBE is about 0.30 and 0.34 eV more negative than the reduction potential of H^+^/H_2_; however, the VBE is about 0.49 and 0.02 eV more negative than the oxidation potential of O_2_/H_2_O, respectively. This means that the reduction ability of H^+^ ions is improved for B_sub_ and remains possible for Ni/Se^2−^, while the VBE position is unsuitable for the oxygen evolution reaction.

## 4. Conclusion

In summary, the screened coulomb hybrid HSE functional was used to study the structural, electronic and optical properties of modified anatase TiO_2_. It was found that the bandgaps are narrowed for all the systems except for the B_int_, B_int+sub_, and Se^4+^ mono-doped and Se/B_int+sub_ co-doped materials with the smallest bandgap calculated for Ni/B_sub_ (2.49 eV). For all the mono- and co-doped materials, the calculated DOS showed new states in the forbidden gap by the incorporation of the dopant into the lattice. The electrons occupying these states are the reason for the enhanced photoactivity of modified anatase under visible light irradiation. The reduction in the bandgaps is associated with the red shift observed for the absorption edge on the Ni, Se^2−^, B_sub_ mono-doped and Ni/B_sub_, Ni/B_int+sub,_ Se/B_sub_, Ni/Se^4+^, Ni/Se^2−^ co-doped systems. The optical response was explored from the absorption coefficient calculations, which indicated a red-shifted absorption edge for all the modified anatase materials. The relative band position determination showed that the Ni, Se^4+^ and Se^2−^ mono-doped in addition to Ni/Se^4+^ co-doped materials are expected to be candidates for water splitting and hydrogen production. In contrast, the Ni/B_int+sub_ and Se/B_int+sub_ co-doped photocatalysts can be used for other energy applications with a visible light response and prolonged lifetime of photogenerated carriers.

## Conflicts of interest

There are no conflicts to declare.

## Supplementary Material

RA-010-D0RA07781J-s001
