# Supplementary material for: Origin of the enhanced photocatalytic activity of (Ni, Se, and B) mono- and co-doped anatase TiO2 materials under visible light: a hybrid DFT study
Source: RSC Adv. 2020 Nov 26;10(70):43092–102. doi: 10.1039/d0ra07781j (PMC9058140; doi:10.1039/d0ra07781j)
Supplement: RA-010-D0RA07781J-s001 [file RA-010-D0RA07781J-s001.pdf]

Electronic Supplementary Material (ESI) for RSC Advances.
This journal is © The Royal Society of Chemistry 2020

Supplementary Information

Origin of the enhanced photocatalytic activity of (Ni, Se, and B) mono- and co-doped anatase TiO_2 materials under visible light: a hybrid DFT study

Hanan H. Ibrahim[a], Adel A. Mohamed[a], Ismail A. M. Ibrahim[a,*]

[a]Department of Chemistry, Faculty of Science, Helwan University, 11795 Cairo, Egypt.

* Corresponding Author:

E-mail address: ismail.ibrahim@science.helwan.edu.eg (Ismail Ibrahim)

Fractional coordinates of the most stable co-doped structures

Ni/B_{sub} co-doped TiO_2

1.01695753293575

11.328000686999998	0.000000000000000	0.000000000000000
0.000000000000000	11.328000686999998	0.000000000000000
0.000000000000000	0.000000000000000	9.4759998321999994

Ni B O Ti

1 1 70 35

Direct

0.3303164252404370	0.4166666570000004	0.3862727494893621
0.4803919689669343	0.4166666570000004	0.3387806771587237
0.8415184951984904	0.4166666570000004	0.3252368537363235
0.0004004640286611	0.0855348826225875	0.1685189431694904
0.0000783262616151	0.9166666870000029	0.8312536392197959
0.1664033206442186	0.9166666870000029	0.6698221440513576
0.1666465226846163	0.0830945778625378	0.3315519818903715
0.0021962931532078	0.2493980373065186	0.4194715683090601
0.3326763340064379	0.0824393717646268	0.5823837958738080
0.1644946038666579	0.0833050560255336	0.9208093848348409
0.1735971883627712	0.2523362611624524	0.0819598101738040
0.9996391917712066	0.0826636870404575	0.5813441224026002
0.3357059627103646	0.2480891409153221	0.4213349566770108
0.1656987725062233	0.2460806941025993	0.6695923362402469
0.9998964626899423	0.2504705486324110	0.8320808297527127
0.9954640312451394	0.4166666570000004	0.1547416957979796

0.9998964626899423	0.5828627643675861	0.8320808297527127
0.1656987725062233	0.5872526188973980	0.6695923362402469
0.1623472061371337	0.4166666570000004	0.3160187047351515
0.6632781581031343	0.2504739085808771	0.4193828363847822
0.6673748389815264	0.0828973921695777	0.5812501716592833
0.5003733407323470	0.0827946023590573	0.9196062998827100
0.8325146286274109	0.2495521510093895	0.0790234594054610
0.4971665023385065	0.2517975965566904	0.0820710601244764
0.8334296052309084	0.0826251416189145	0.9181790412548824
0.1666465226846163	0.7502387581374648	0.3315519818903715
0.0004004640286611	0.7477984533774154	0.1685189431694904
0.3334537255862152	0.0830359524750746	0.1686279271944827
0.6664600179381432	0.9166666870000029	0.8310126163923257
0.8334092511831245	0.9166666870000029	0.6690365045335968
0.5005051069061998	0.0827032939998862	0.3309897681816434
0.0021962931532078	0.5839352756934785	0.4194715683090601
0.3326763340064379	0.7508939642353757	0.5823837958738080
0.1644946038666579	0.7500282799744690	0.9208093848348409
0.1735971883627712	0.5809970518375449	0.0819598101738040
0.8332832237942668	0.0841052509121837	0.3308222769304063
0.4999669868783796	0.9166666870000029	0.6695305433635707
0.3329762346949731	0.9166666870000029	0.8331517278514036
0.6665769849153506	0.0842372627119526	0.1684603274627395
0.9996391917712066	0.7506696489595454	0.5813441224026002
0.3357059627103646	0.5852441720846752	0.4213349566770108

0.4999180336614180	0.2490523016660107	0.6687877977562431
0.3329597562135392	0.2459921742634936	0.8331889846584031
0.1679897871905386	0.4166666570000004	0.9140645763620077
0.1667140513475854	0.9166666870000029	0.0829054139259642
0.3339030398887864	0.9166666870000029	0.4198421944367530
0.0026808038887327	0.4166666570000004	0.5800861461440090
0.6664235727612051	0.2476454127704212	0.8314679458200637
0.8337524842451610	0.2486224300528579	0.6678539819339706
0.3270433349614346	0.4166666570000004	0.6009314816460090
0.0003321484628349	0.9166666870000029	0.4181268228053607
0.6664235727612051	0.5856879002295762	0.8314679458200637
0.8337524842451610	0.5847108829471390	0.6678539819339706
0.6632781581031343	0.5828594044191201	0.4193828363847822
0.6673748389815264	0.7504359438304252	0.5812501716592833
0.5003733407323470	0.7505387336409453	0.9196062998827100
0.8325146286274109	0.5837811619906080	0.0790234594054610
0.4999180336614180	0.5842810113339864	0.6687877977562431
0.3329597562135392	0.5873411387365036	0.8331889846584031
0.6732142024179788	0.4166666570000004	0.1702987722268483
0.4971665023385065	0.5815357164433068	0.0820710601244764
0.8334296052309084	0.7507081943810883	0.9181790412548824
0.8332832237942668	0.7492280850878188	0.3308222769304063
0.6665769849153506	0.7490960732880501	0.1684603274627395
0.8307807296254262	0.4166666570000004	0.9147666652321507
0.4994600182668516	0.9166666870000029	0.0822609778983513

0.6663359089699450	0.9166666870000029	0.4185037258691292
0.6704315444322833	0.4166666570000004	0.5868702380265658
0.3334537255862152	0.7502973835249277	0.1686279271944827
0.5005051069061998	0.7506300420001165	0.3309897681816434
0.8338978637819873	0.9166666870000029	0.0818310643642520
0.4987329783299383	0.4166666570000004	0.9100674910261928
0.0016116196048755	0.0828814196036208	0.3742309518516133
0.0015470983470307	0.9166666870000029	0.6257308331632074
0.1669479076533869	0.9166666870000029	0.8759749935432696
0.1674667973891612	0.0832350886935808	0.1255699661706939
0.0012945542893149	0.2479257714976060	0.6252870764951122
0.3337861541765624	0.0858811056247751	0.3785958725659133
0.1674214153936746	0.2501207311414693	0.8749151432327584
0.3321369475728700	0.2532945875612699	0.6227432515157024
0.0043100054001597	0.4166666570000004	0.3789151829257197
0.0012945542893149	0.5854075415023912	0.6252870764951122
0.1674214303936722	0.5832125818585280	0.8749151432327584
0.1477021495774397	0.4166666570000004	0.1223730573279771
0.6663624853672251	0.2470699655282155	0.6277464883618428
0.6668599621518690	0.0827392877863700	0.3737430026439422
0.4976953747693289	0.0825763881587709	0.1263142113373190
0.8333543238580287	0.2480123460984955	0.8730113391545157
0.4965561914811097	0.2467993857512243	0.8723929026367175
0.8328508690686032	0.0830417645825340	0.1251716831949348
0.1674667973891612	0.7500982473064215	0.1255699661706939

0.0016116196048755	0.7504519163963820	0.3742309518516133
0.6671116346633141	0.9166666870000029	0.6261109409269299
0.8328769773765247	0.9166666870000029	0.8740274688399418
0.3337861541765624	0.7474522303752273	0.3785958725659133
0.4978663272571621	0.9166666870000029	0.8744041027641234
0.3332196133048583	0.9166666870000029	0.6222938466829171
0.3321369475728700	0.5800387254387273	0.6227432515157024
0.6663624853672251	0.5862633474717815	0.6277464883618428
0.8333543238580287	0.5853209669015017	0.8730113391545157
0.5234550488498687	0.4166666570000004	0.1081135278530313
0.6668599621518690	0.7505940482136325	0.3737430026439422
0.4976953747693289	0.7507569478412319	0.1263142113373190
0.8323623548827459	0.4166666570000004	0.1172014224516298
0.4965561914811097	0.5865339272487731	0.8723929026367175
0.6797021527347569	0.4166666570000004	0.3878782029589384
0.8328508690686032	0.7502915714174685	0.1251716831949348

Ni/$B_{int+Sub}$ co-doped TiO_2

1.01997553460240

11.3280000686999998 0.0000000000000000 0.0000000000000000

0.0000000000000000 11.3280000686999998 0.0000000000000000

0.0000000000000000 0.0000000000000000 9.4759998321999994

Ni B O Ti

1 2 70 35

Direct

0.6617515429343529 0.4175549629787163 0.3781638781922014

0.8343627771738203 0.7524589675392055 0.5256366123557670

0.8170816608413469 0.4204937433367473 0.3398523316936477

-0.0000213337601212 0.0830215367086370 0.1695417404842634

0.0004232383592875 0.9173990662066509 0.8321369499214576

0.1651927637198798 0.9110969892479098 0.6673582705924623

0.1673219036082844 0.0848798355493109 0.3296395579465775

0.9955541659174921 0.2498977686883176 0.4160597030152597

0.3329212727107136 0.0811998759259699 0.5814952760326553

0.1670307276270099 0.0826261500635727 0.9159738137968584

0.1670566097457014 0.2496620907726433 0.0772469548095345

0.0049426021839875 0.0857219867292029 0.5808772790749025

0.3355014969126618 0.2489174443311124 0.4199044978906324

0.1679983322515399 0.2478807711423704 0.6678211045002271

0.9995411512172320 0.2493558782291619 0.8307553947149835

0.0077411588242286 0.4169748440185562 0.1677242794894608

0.9983380638094764 0.5830285731988830 0.8307039073839063

0.1648928253288267	0.5904234953335882	0.6664623435637682
0.1773659322580682	0.4167979139234181	0.3229045080107530
0.6673334464305424	0.2429124810694854	0.4214337625806087
0.6629820296725812	0.0830742175352996	0.5852014879380032
0.4984670815575113	0.0825089911830414	0.9184632255989339
0.8318809506793465	0.2506570888549393	0.0831982387530065
0.5056005916258508	0.2517922549966292	0.0798840085044551
0.8337922162168232	0.0848203842029523	0.9208638112488440
0.1707106008703390	0.7502608690112403	0.3289320181944714
0.9896760320759410	0.7483837129388865	0.1782450506520127
0.3339502778660750	0.0837092354640128	0.1673055117613697
0.6671568605560533	0.9169831558317530	0.8339213486703825
0.8346936598223038	0.9403827969537516	0.6722096873022616
0.4989791588327423	0.0820415747173572	0.3295519651898425
0.0087779764576192	0.5866420366376908	0.4247483067347393
0.3324544420485182	0.7504021144450708	0.5782285263535348
0.1666771840987259	0.7505017013855387	0.9166266088425776
0.1641656351692110	0.5824757352933385	0.0769034210582483
0.8342143558195424	0.0761216011326668	0.3324443130451916
0.5021461737142836	0.9090415067219050	0.6686044109146416
0.3334299790287507	0.9175074119540824	0.8292632723900650
0.6673316078081958	0.0831032556132765	0.1696269011767024
0.9457974920710045	0.7520658913302242	0.5836352479224638
0.3362017300288069	0.5841504392176777	0.4177709086362746
0.4984356260429333	0.2455949976602067	0.6695384516088709

0.3333646794043694	0.2507214970120630	0.8322110739936057
0.1653793169557160	0.4167404241375937	0.9121595029397553
0.1659571654904761	0.9179221048994223	0.0800563974396453
0.3325506788941826	0.9169631809247191	0.4178699988277392
0.0084689949459504	0.4139169762622635	0.5859111895384324
0.6659352351615843	0.2453071519797380	0.8321260095462621
0.8322431947438791	0.2496060706747708	0.6684371469443333
0.3345500003137787	0.4177038803989060	0.5799131833622286
0.0088462335209508	0.9142940307903576	0.4246747279773551
0.3311215316869283	0.4196130746278006	0.1518919003819983
0.6663623372190526	0.5879177402794419	0.8315486873188676
0.8322523400539188	0.5630232345370965	0.6674804086574819
0.4954024158574412	0.4155354279222262	0.3151152124704825
0.6490778735679413	0.5986003773619973	0.4327958596738067
0.7260989092299077	0.7564027965610871	0.5877313066871522
0.4980693639917689	0.7502374083651518	0.9191603456800967
0.8321754311597687	0.5802041758596804	0.0791041608745405
0.4992328334029549	0.5918507906511374	0.6696078274170468
0.3325230305254244	0.5817346837617918	0.8301804506261995
0.5088158123919453	0.5813656666636375	0.0806054302716024
0.8331780115403030	0.7500389809287767	0.9147030693081182
0.8336711305254257	0.7497154845123267	0.3829929392858141
0.6768630121280549	0.7500112948667115	0.1776547474282738
0.8268554956137970	0.4143377526111773	0.9107582067246685
0.5021958035584746	0.9170658910888057	0.0810461924433450

0.6571003639042867	0.9141177099081360	0.4273198769498124
0.6527398368191274	0.4128862996924145	0.5958818441887770
0.3343728020937140	0.7478236642839851	0.1616349346093148
0.4956640114246310	0.7551928299167560	0.3276478577582642
0.8343284600254766	0.9171641141860033	0.0803762183655322
0.5029029211610239	0.4170770203730033	0.9119345068161216
0.0030438307274823	0.0821643555480272	0.3701436673415305
0.0002782626311954	0.9303781189644238	0.6283224947447070
0.1677099369237000	0.9175148971752780	0.8746446794452249
0.1683032169858954	0.0828856662242361	0.1222663212949445
0.0005606901139324	0.2496883872084823	0.6303400251142782
0.3345838839931596	0.0834684636214981	0.3742359066301609
0.1687858208513153	0.2492186605042700	0.8735139641734099
0.3333016238849905	0.2476227400332815	0.6252756591285279
0.0095743377908356	0.4117180406530888	0.3761124225177644
0.9982996086853542	0.5699536798113602	0.6279670513742275
0.1673903274073199	0.5825530743919599	0.8724541335938754
0.1680564365013768	0.4176011647749353	0.1176892919902331
0.6642455592781126	0.2496653334887336	0.6249467679231077
0.6654692948548984	0.0872527109206809	0.3733532176527422
0.4991735368087206	0.0829453142168008	0.1235318449771628
0.8304778476633952	0.2492035794677225	0.8709058301492686
0.5006596652455778	0.2507879721301989	0.8740112987403562
0.8322237758846696	0.0836827584470909	0.1285004814657058
0.1709529843006124	0.7497419682892337	0.1175904166583541

0.0132935219704367	0.7487725981763769	0.3685932992735116
0.6663752832806777	0.9278664830968153	0.6282251315977245
0.8337162235754892	0.9252040991688519	0.8736586527506239
0.3346594561276149	0.7503591855664986	0.3778330990449524
0.5004723869606544	0.9178348785311659	0.8753250517175950
0.3326387524054783	0.9171443304788833	0.6247366552537592
0.3324583795537495	0.5832489622971920	0.6250859830070459
0.3375450417071175	0.4169818718599909	0.3779899277547763
0.6629500119937446	0.5667989363885685	0.6245388246066537
0.8309697908797947	0.5762840091939405	0.8700992740468260
0.4832069385703751	0.4168685052465380	0.1217848168521556
0.6541301278319033	0.7526304917436062	0.3707675364616926
0.4943095584202358	0.7493585455666039	0.1189285120201973
0.8547257967174243	0.4145749843884617	0.1162293898994659
0.5020848834343294	0.5822150114287277	0.8737294868125495
0.8318888145686145	0.7495263538825738	0.1046787809313852

Se/B_{sub} co-doped TiO_2

1.01928471735426

11.3280000686999998 0.0000000000000000 0.0000000000000000

0.0000000000000000 11.3280000686999998 0.0000000000000000

0.0000000000000000 0.0000000000000000 9.4759998321999994

Se B O Ti

1 1 71 35

Direct

0.3284695902278227 0.4166666570000004 0.3697158209878578

0.4967064582027374 0.4166666570000004 0.3728133635656856

0.8395342470813402 0.4166666570000004 0.3174224831044102

0.9993544018780622 0.0846741362361418 0.1693313120256942

0.0009660529708891 0.9166666870000029 0.8308057502410821

0.1672879672244053 0.9166666870000029 0.6699730021971222

0.1663870779015950 0.0857381376603768 0.3314436144077126

0.9943827666113580 0.2476695184741456 0.4224391205381299

0.3323264848589852 0.0814850095029456 0.5809882190585207

0.1664871592979451 0.0822310409029156 0.9194385897004697

0.1670100748384127 0.2498175231250937 0.0779147162021556

0.0022698886354736 0.0817154141449788 0.5819697428851214

0.3441617292485842 0.2474547343470098 0.4238304950460883

0.1672484968155031 0.2471852325770354 0.6685166024274997

0.0006454823507976 0.2499097539522842 0.8320994761260327

0.9906225733691161 0.4166666570000004 0.1230968872934779

0.0006454823507976 0.5834235590477128 0.8320994761260327

0.1672484968155031	0.5861480804229616	0.6685166024274997
0.1354090149215960	0.4166666570000004	0.3090682072490302
0.6637331529732499	0.2504318820316984	0.4194115903066872
0.6682027008265686	0.0825894618016065	0.5803561840324823
0.5025741902354556	0.0820675755266108	0.9194988752824587
0.8274044236991881	0.2483200366319416	0.0769310094356266
0.4991469335242681	0.2495849909025135	0.0816045733298011
0.8350009685168978	0.0818458706529778	0.9179435196811203
0.1663870779015950	0.7475951983396261	0.3314436144077126
0.9993544018780622	0.7486591997638609	0.1693313120256942
0.3326918445406514	0.0839182499296839	0.1688439863440786
0.6675790460124803	0.9166666870000029	0.8307598278378125
0.8341777222407678	0.9166666870000029	0.6687434883124596
0.5001253233171740	0.0809076523570265	0.3303506386031091
0.9943827666113580	0.5856637945258447	0.4224391205381299
0.3323264848589852	0.7518483264970570	0.5809882190585207
0.1664871592979451	0.7511022950970865	0.9194385897004697
0.1670100748384127	0.5835157898749039	0.0779147162021556
0.8323382473623281	0.0827784927494029	0.3303391353595871
0.5014037556348164	0.9166666870000029	0.6695967508494970
0.3344259769627127	0.9166666870000029	0.8315479001947590
0.6661282007118269	0.0825564569152991	0.1688652909346986
0.0022698886354736	0.7516179218550231	0.5819697428851214
0.3441617292485842	0.5858785786529872	0.4238304950460883
0.5025794487807871	0.2485566253038916	0.6700221077302844

0.3352794665974703	0.2440680607253450	0.8340972345291655
0.1711686254385073	0.4166666570000004	0.9092944141295265
0.1660964043643387	0.9166666870000029	0.0824974221794234
0.3335992543137116	0.9166666870000029	0.4180376870279184
0.0001148773162211	0.4166666570000004	0.5840561962100020
0.6672223361814120	0.2461154171072695	0.8310274029197872
0.8344729348828223	0.2450442653945314	0.6686068900275222
0.3340680781526972	0.4166666570000004	0.6113759075876471
0.9993431717926262	0.9166666870000029	0.4182935078334803
0.3343165653172174	0.4166666570000004	0.1837826785778399
0.6672223361814120	0.5872178958927271	0.8310274029197872
0.8344729348828223	0.5882890476054652	0.6686068900275222
0.6637331529732499	0.5829014309682988	0.4194115903066872
0.6682027008265686	0.7507438741983963	0.5803561840324823
0.5025741902354556	0.7512657604733919	0.9194988752824587
0.8274044236991881	0.5850132763680557	0.0769310094356266
0.5025794487807871	0.5847766876961055	0.6700221077302844
0.3352794665974703	0.5892652522746521	0.8340972345291655
0.6627638379428554	0.4166666570000004	0.1706077180035634
0.4991469335242681	0.5837483220974835	0.0816045733298011
0.8350009685168978	0.7514874653470245	0.9179435196811203
0.8323382473623281	0.7505548432506001	0.3303391353595871
0.6661282007118269	0.7507768790847036	0.1688652909346986
0.8266508984936702	0.4166666570000004	0.9090823049071420
0.4984422595163444	0.9166666870000029	0.0816963597207319

0.6663543051927567	0.9166666870000029	0.4187226432827096
0.6789819955871914	0.4166666570000004	0.5870224336017535
0.3326918445406514	0.7494150860703190	0.1688439863440786
0.5001253233171740	0.7524256836429838	0.3303506386031091
0.8330388255221403	0.9166666870000029	0.0809213167203628
0.5018005953932104	0.4166666570000004	0.9146950214914193
0.9984993712302681	0.0828667536669084	0.3738586918032037
0.0023896327353367	0.9166666870000029	0.6256756940015759
0.1680643366269242	0.9166666870000029	0.8755722564181084
0.1667981078809373	0.0834866763919153	0.1246148835150907
0.0025441771029168	0.2457506782679956	0.6264408386294219
0.3319286100285454	0.0817944013322644	0.3779441012273457
0.1683916631512407	0.2496062521317345	0.8741397349298251
0.3357155099690552	0.2533590261970109	0.6290227097544380
0.9928764730869543	0.4166666570000004	0.3870143414665178
0.0025441771029168	0.5875826347320018	0.6264408386294219
0.1683916781512384	0.5837270608682626	0.8741397349298251
0.1493359626624400	0.4166666570000004	0.1154651447930515
0.6684789082157400	0.2470583310564944	0.6271334689858062
0.6657857717322037	0.0822363272644702	0.3735464802995668
0.4978725026898911	0.0820626768383475	0.1266416072161266
0.8343787194842890	0.2478825733654747	0.8729760520503659
0.5011700845037970	0.2460700354301024	0.8749794852517009
0.8320135134993887	0.0827802656751822	0.1239872427803707
0.1667981078809373	0.7498466596080873	0.1246148835150907

0.9984993712302681	0.7504665823330943	0.3738586918032037
0.6682340202636652	0.9166666870000029	0.6253191699449301
0.8350079661065964	0.9166666870000029	0.8735194245194915
0.3319286100285454	0.7515389346677380	0.3779441012273457
0.5021464991571976	0.9166666870000029	0.8743100522723866
0.3339462014124961	0.9166666870000029	0.6265283665100724
0.3357155099690552	0.5799742868029855	0.6290227097544380
0.6684789082157400	0.5862749819435027	0.6271334689858062
0.8343787194842890	0.5854507396345220	0.8729760520503659
0.5093367591396607	0.4166666570000004	0.1133264927297943
0.6657857717322037	0.7510970087355328	0.3735464802995668
0.4978725026898911	0.7512706591616551	0.1266416072161266
0.8315324306530331	0.4166666570000004	0.1109509536244732
0.5011700845037970	0.5872632775698946	0.8749794852517009
0.6823064658133500	0.4166666570000004	0.3863777638587181
0.8320135134993887	0.7505530703248200	0.1239872427803707

Se/$B_{int+sub}$ co-doped TiO_2

1.02352557834097

11.3280000686999998 0.0000000000000000 0.0000000000000000

0.0000000000000000 11.3280000686999998 0.0000000000000000

0.0000000000000000 0.0000000000000000 9.4759998321999994

Se B O Ti

1 2 71 35

Direct

0.7016059452869602 0.4238087160855172 0.3252817762038828

0.8356390674295729 0.7544070107871852 0.5298179137406628

0.8509409180222754 0.4676886580639542 0.3343822381636401

0.4878686959993580 0.4148808329060435 0.3128847696006149

0.9996684235467096 0.0819837533687646 0.1710040193070326

0.0017421715934430 0.9156433507176222 0.8340871588509083

0.1652494391144112 0.9112970872796103 0.6687931761946173

0.1665357686072240 0.0832339468900555 0.3291990354846327

0.9999575941689102 0.2492032054452250 0.4172775964435672

0.3333760667962962 0.0821981265104434 0.5814346610658887

0.1671102715714392 0.0813905747276170 0.9172556704821663

0.1670003590950714 0.2470240830838588 0.0769221457270673

0.0053776238452858 0.0864995095851926 0.5817677186718764

0.3313081554102892 0.2492033450933889 0.4191007228521750

0.1672763226170287 0.2496440393153866 0.6674139521172674

0.9996007051790298 0.2451519429702273 0.8281468493192634

0.0026588140399117 0.4020800867329399 0.1681361663116140

0.9990752351980966	0.5863309867156087	0.8288166839190033
0.1657974639088589	0.5889815070471983	0.6691430438574145
0.1747234874821016	0.4278487666838966	0.3200138951812466
0.6582407729837533	0.2298449690957809	0.4298210063479551
0.6645373478645598	0.0781032204775878	0.5937729781641025
0.4973207677331176	0.0809603679384592	0.9184714617056133
0.8330667684064698	0.2457873353012853	0.0785932003067006
0.4975510888378133	0.2474073550817663	0.0783857311199315
0.8332311382701234	0.0822889575269337	0.9214903781434605
0.1720674881540251	0.7557261986087505	0.3271570937983382
0.9895808605280031	0.7490565964776748	0.1819235319381127
0.3318984166841601	0.0807129958952435	0.1681325457565298
0.6667229636346284	0.9159892868722871	0.8369536225106020
0.8375029687999398	0.9435767320309754	0.6772338254775723
0.4965096268393407	0.0791631306764889	0.3289732261535983
0.0234551273011173	0.5947142711068507	0.4340453061586684
0.3332061125305041	0.7502691511009680	0.5789317234182728
0.1676780502702953	0.7499281730774520	0.9186108963749887
0.1586724463514196	0.5836487253649109	0.0780838543394090
0.8332319467917864	0.0754832690898139	0.3327921219606689
0.5006999597458929	0.9082823617129755	0.6703982162107219
0.3327570297674590	0.9175224941104935	0.8298468254260444
0.6656629913366096	0.0809657641452612	0.1710999086280375
0.9478588858919301	0.7565539323205858	0.5855471583077454
0.3367112522695988	0.5860680970162233	0.4184075489900435

0.4966687822545370	0.2477706582391314	0.6690913312231618
0.3316078178490610	0.2492366238773825	0.8299357875894963
0.1624628502000234	0.4158747760946942	0.9136002753426247
0.1634567485721821	0.9154445264965105	0.0801415938660752
0.3310368277612381	0.9175411324086379	0.4212103494211130
0.0065197617086259	0.4139866655111843	0.5848310071643669
0.6647061743991357	0.2435286109672140	0.8342353584380902
0.8322510079889041	0.2467729990504905	0.6670095323668710
0.3324899532741620	0.4181980046761674	0.5777585981907538
0.0088105043171183	0.9163960595552104	0.4268919741310279
0.3281502901977004	0.4141549034308376	0.1514921937455264
0.6664345526040393	0.5852567254973912	0.8346263838857579
0.8335153994616902	0.5682777087063482	0.6648309002905667
0.6314094058371608	0.6167611971042055	0.4529047315365243
0.7309671042297197	0.7645068706988019	0.5951355687282293
0.4977697590732511	0.7495836858006493	0.9182493477989858
0.8425445546975879	0.5828553416181336	0.0704706383223081
0.4960169308822160	0.5852939982569240	0.6748999926808928
0.3304470258491430	0.5813371013836350	0.8318076241635721
0.6704006440339422	0.4223171192946947	0.1393478963553064
0.4963870093370266	0.5834069518969811	0.0802796663388181
0.8317075989795556	0.7512804734577795	0.9140308027956040
0.8332382192340150	0.7457063815284979	0.3875881474490439
0.6775404861526292	0.7442099341119368	0.1783166413173566
0.8359650660047688	0.4130028585209654	0.9071134384463380

0.5006972008621849	0.9151489703159310	0.0800452157699486
0.6540402605937381	0.9138531356350453	0.4303619496172256
0.6632135615016848	0.4117284826358334	0.6030653670920907
0.3307236650264797	0.7460233519479209	0.1595516222578034
0.4931301987470209	0.7627697058482451	0.3212072456246101
0.8342498738834423	0.9159124190105634	0.0822582045786576
0.4990063681925470	0.4153252070527517	0.9118308190888863
0.0029120321605988	0.0819376183286487	0.3700496450338770
0.0000016999557867	0.9310497227299903	0.6313734709193323
0.1679291661304933	0.9174840433242034	0.8754227613562822
0.1678279685439659	0.0828848175625951	0.1232186304317134
0.9973449369056957	0.2481135870583077	0.6318644479787798
0.3342916196690146	0.0822996221852860	0.3750043933023564
0.1683153686028749	0.2475670258713836	0.8724042279604555
0.3312727177345901	0.2508724508614351	0.6225766023287391
0.0211945267048380	0.4096141092994228	0.3806502549286699
0.9985103274833569	0.5717141653525510	0.6287808301015144
0.1669676905943495	0.5825331244539963	0.8733187356394192
0.1650529719356247	0.4148118885492028	0.1157660654040205
0.6603710256633992	0.2499919190395873	0.6208421879253113
0.6634095989755023	0.0790500341632015	0.3766823762021258
0.4981935337657361	0.0819876937357638	0.1234001287766289
0.8258537932148736	0.2437400858983370	0.8685068646198003
0.4980534204258532	0.2490886392270112	0.8737348554528983
0.8307214359545435	0.0801813102050494	0.1300466596579041

0.1705159228230116	0.7485145352161464	0.1173830792158454
0.0154456888679426	0.7514226994150208	0.3717290322208858
0.6661629465694976	0.9257725938564950	0.6361942287224480
0.8347017378603259	0.9232973535363985	0.8751363624537031
0.3354236908228239	0.7499951510326602	0.3793322742906444
0.4988536168810614	0.9174629930424133	0.8765548506919330
0.3330872938978652	0.9179759303366968	0.6253338868387801
0.3322347365121057	0.5820317777071534	0.6249489366897900
0.6612750347735804	0.5640733500908665	0.6308062579908633
0.8317292626042703	0.5791586275332197	0.8701713366198439
0.4884618765017459	0.4134457817257851	0.1183645332496718
0.6533833460413828	0.7566405290225735	0.3727757599287947
0.4921511285690631	0.7475055687897759	0.1173712693809381
0.8447938352906987	0.4079725994463462	0.1047943637604404
0.4981039560505673	0.5815467693815907	0.8761385077951859
0.8317924213369660	0.7471199023536478	0.1049420323146919
0.3387510495645866	0.4171263579981137	0.3707085894664002

Ni/Se^{2-} co-doped TiO_2

1.01976996928720

11.3280000686999998 0.0000000000000000 0.0000000000000000

0.0000000000000000 11.3280000686999998 0.0000000000000000

0.0000000000000000 0.0000000000000000 9.4759998321999994

Ni Se O Ti

1 1 70 35

Direct

0.3342533803415238 0.4166666570000004 0.4132540565901957

0.3161445283810591 0.4166666570000004 0.1814059902594802

0.3360856443933697 0.4166666570000004 0.6109117312322393

0.5137037876438020 0.4166666570000004 0.3208036055436378

-0.0001249699776490 0.0789120930319678 0.1694707334462932

0.9994677081502287 0.9166666870000029 0.8293482013904412

0.1659440397138406 0.9166666870000029 0.6668876423641841

0.1669897435492604 0.0840582991595851 0.3286412384098656

0.0055949612485246 0.2522044300469072 0.4184202033958865

0.3356835015376933 0.0809774868432226 0.5817200058470082

0.1706184868064142 0.0791685425663283 0.9147266392244232

0.1461644220152185 0.2460326411524371 0.0670757588394620

0.9976386203673191 0.0825079685429220 0.5788802304315139

0.3303551459467926 0.2495442596826422 0.4296590965521403

0.1666239728205837 0.2437149491460216 0.6655774668958585

0.9987255747480840 0.2426084314871728 0.8271148117607875

-0.0000988161313254 0.4166666570000004 0.1885956512631298

0.9987255747480840	0.5907248815128245	0.8271148117607875
0.1666239728205837	0.5896183638539755	0.6655774668958585
0.6677813382940418	0.2478381884989595	0.4218195883506268
0.6676068509234550	0.0819587228800447	0.5818763152620476
0.4997775496805831	0.0820717301805301	0.9192384153940897
0.8393819196421128	0.2491404915262180	0.0801523545744239
0.5128859230288449	0.2484366528316629	0.0786035190299713
0.8317662822035070	0.0824624415228254	0.9189952270912394
0.1669897435492604	0.7492750368404176	0.3286412384098656
-0.0001249699776490	0.7544212429680349	0.1694707334462932
0.3326353785374251	0.0912887264174003	0.1670806779819166
0.6667484042355268	0.9166666870000029	0.8311613039365396
0.8333302705138881	0.9166666870000029	0.6690236339147875
0.4995287913936741	0.0847869046214281	0.3306062780242487
0.0055949612485246	0.5811288829530905	0.4184202033958865
0.3356835015376933	0.7523558491567804	0.5817200058470082
0.1706184868064142	0.7541647934336739	0.9147266392244232
0.1461644220152185	0.5873006718475603	0.0670757588394620
0.8343226781441471	0.0852296052275511	0.3313834414818154
0.5010550342983029	0.9166666870000029	0.6689106498723782
0.3341947379430246	0.9166666870000029	0.8291449233349778
0.6679067535105642	0.0808858665576639	0.1702590678958839
0.9976386203673191	0.7508253674570806	0.5788802304315139
0.3303551459467926	0.5837890533173548	0.4296590965521403
0.5018585276769064	0.2450222907013550	0.6712614937231721

0.3338049409943185	0.2466486644359067	0.8369799307256964
0.1579076001292177	0.4166666570000004	0.8938640746076392
0.1667765610189236	0.9166666870000029	0.0801709989460085
0.3328400471796079	0.9166666870000029	0.4169184945514079
0.9979512179115787	0.4166666570000004	0.5878045149845308
0.6689128486585025	0.2500499069254101	0.8322368455758037
0.8329623789088167	0.2493864852390200	0.6673813058231796
0.0013916709080983	0.9166666870000029	0.4172487521630980
0.6689128486585025	0.5832834060745873	0.8322368455758037
0.8329623789088167	0.5839468277609771	0.6673813058231796
0.6677813382940418	0.5854951245010374	0.4218195883506268
0.6676068509234550	0.7513746131199578	0.5818763152620476
0.4997775496805831	0.7512616058194723	0.9192384153940897
0.8393819196421128	0.5841928214737792	0.0801523545744239
0.8276047738069756	0.4166666570000004	0.3413130507690384
0.5018585276769064	0.5883110222986422	0.6712614937231721
0.3338049409943185	0.5866846485640906	0.8369799307256964
0.6807544858954530	0.4166666570000004	0.1647412529912858
0.5128859230288449	0.5848966601683342	0.0786035190299713
0.8317662822035070	0.7508708944771771	0.9189952270912394
0.8343226781441471	0.7481037307724516	0.3313834414818154
0.6679067535105642	0.7524474694423388	0.1702590678958839
0.8410673916459425	0.4166666570000004	0.9196899088287580
0.4972455860628538	0.9166666870000029	0.0823523433843374
0.6665838298484104	0.9166666870000029	0.4187757920527385

0.6622375605726908	0.4166666570000004	0.5852642976984187
0.3326353785374251	0.7420446095826021	0.1670806779819166
0.4995287913936741	0.7485464313785748	0.3306062780242487
0.8339912186524212	0.9166666870000029	0.0805410988246010
0.5018301750405989	0.4166666570000004	0.9160640546185023
0.0019894833520840	0.0825673211438405	0.3733058229607221
0.9980794201326734	0.9166666870000029	0.6248564446178975
0.1670609085934463	0.9166666870000029	0.8689775303308249
0.1653431306536220	0.0821633755393375	0.1235347003601042
0.0011027824399793	0.2468131173895886	0.6256856383344979
0.3338729555439748	0.0872289419411089	0.3781388054693169
0.1691805080792742	0.2398883686161057	0.8669920003900931
0.3366002473414719	0.2505512656174629	0.6268783267007340
0.9752013886197914	0.4166666570000004	0.3893748085661252
0.0011027824399793	0.5865201956104080	0.6256856383344979
0.1691805230792719	0.5934449443838912	0.8669920003900931
0.1359341113122363	0.4166666570000004	0.0816215546275761
0.6678255940591262	0.2472465305175325	0.6253272720086254
0.6671849270653066	0.0827703346954745	0.3746692783901758
0.4978000980237638	0.0833026958645267	0.1254771910182420
0.8341040456473416	0.2458349963457062	0.8734953421755731
0.5018540693044133	0.2480847524535340	0.8755580502941691
0.8324032755238727	0.0823620241899291	0.1265451293567433
0.1653431306536220	0.7511699604606650	0.1235347003601042
0.0019894833520840	0.7507660148561620	0.3733058229607221

0.6658939051941820	0.9166666870000029	0.6260903561929813
0.8317092641515921	0.9166666870000029	0.8745039431820882
0.3338729555439748	0.7461043940588937	0.3781388054693169
0.4975174065502112	0.9166666870000029	0.8746041517186063
0.3347385385293239	0.9166666870000029	0.6208335032266925
0.3366002473414719	0.5827820473825344	0.6268783267007340
0.6678255940591262	0.5860867824824648	0.6253272720086254
0.8341040456473416	0.5874983166542909	0.8734953421755731
0.5236921116229954	0.4166666570000004	0.1177617377730933
0.6671849270653066	0.7505630013045282	0.3746692783901758
0.4978000980237638	0.7500306401354759	0.1254771910182420
0.8376099926101519	0.4166666570000004	0.1172478082192950
0.5018540693044133	0.5852485605464630	0.8755580502941691
0.6595601621406112	0.4166666570000004	0.3849957460297586
0.8324032755238727	0.7509713118100734	0.1265451293567433

Ni/Se^{4+} co-doped TiO_2

1.01636876247283

11.3280000686999998 0.0000000000000000 0.0000000000000000

0.0000000000000000 11.3280000686999998 0.0000000000000000

0.0000000000000000 0.0000000000000000 9.4759998321999994

Ni Se O Ti

1 1 71 34

Direct

0.3329100038601829 0.4166666570000004 0.3861778794978776

0.9944277302614682 0.4166666570000004 0.3965124423486933

0.5075139342664459 0.4166666570000004 0.3313177244847840

0.9992983459886807 0.0828144914215563 0.1687026781485796

0.9990197575531222 0.9166666870000029 0.8308029301509563

0.1666988143649081 0.9166666870000029 0.6678473861487015

0.1650936667347145 0.0823118907435643 0.3295596879666651

0.9959813275466356 0.2478063762271714 0.4258166967079680

0.3345941480030641 0.0831143905081490 0.5817099274655833

0.1657545534259129 0.0811676953230852 0.9151789079970075

0.1679876878130945 0.2469172562422191 0.0753698029772185

0.9998508148340820 0.0807669066172993 0.5832725370965911

0.3255873723655688 0.2480816932210107 0.4195853851352935

0.1647156711553347 0.2488944083693213 0.6665949700171444

-0.0002366902124215 0.2493575107706771 0.8332627135378461

0.0119413336292726 0.4166666570000004 0.1418731122384586

-0.0002366902124215 0.5839758022293199 0.8332627135378461

0.1647156711553347	0.5844389046306758	0.6665949700171444
0.6709055725815929	0.2501521865630872	0.4195129217729603
0.6648140190628511	0.0831401894715762	0.5811154647578111
0.5003976460014705	0.0838270536961191	0.9204618057247105
0.8354877506260885	0.2493797203235069	0.0817394168950703
0.4997921259292349	0.2516980053252061	0.0833775182083126
0.8315339851976534	0.0828485920066711	0.9185676182451348
0.1650936667347145	0.7510214452564383	0.3295596879666651
0.9992983459886807	0.7505188445784459	0.1687026781485796
0.3330222745721897	0.0832600263466243	0.1678527978281267
0.6659887594410344	0.9166666870000029	0.8314027379198321
0.8324256428509829	0.9166666870000029	0.6685986339909814
0.4997719931992277	0.0858848411032791	0.3319977041538046
0.9959813275466356	0.5855269367728252	0.4258166967079680
0.3345941480030641	0.7502189454918536	0.5817099274655833
0.1657545534259129	0.7521656406769176	0.9151789079970075
0.1679876878130945	0.5864160567577776	0.0753698029772185
0.8325232444214136	0.0819098238356913	0.3309285629405024
0.4999191461085991	0.9166666870000029	0.6692387536266179
0.3337087373777733	0.9166666870000029	0.8320114338415202
0.6661091849752491	0.0836912626065268	0.1684920622261497
0.9998508148340820	0.7525664293827036	0.5832725370965911
0.3255873723655688	0.5852516197789863	0.4195853851352935
0.5005022597400568	0.2513012849370107	0.6685491270827090
0.3337279365283514	0.2452460154168104	0.8304446646694393

0.1783416849721335	0.4166666570000004	0.9059262481738130
0.1666134779929364	0.9166666870000029	0.0800383565890682
0.3331005727523768	0.9166666870000029	0.4190723742711286
0.9845197945777230	0.4166666570000004	0.5782336614695132
0.6657817735589897	0.2517265137464010	0.8330107940802394
0.8321477909965048	0.2446576310796625	0.6712551837694777
0.3316153245548701	0.4166666570000004	0.5882244712193502
0.9984568869711324	0.9166666870000029	0.4181663942749552
0.3208601772804237	0.4166666570000004	0.1826994971525950
0.6657817735589897	0.5816067992535959	0.8330107940802394
0.8321477909965048	0.5886756819203344	0.6712551837694777
0.6709055725815929	0.5831811264369100	0.4195129217729603
0.6648140190628511	0.7501931465284267	0.5811154647578111
0.5003976460014705	0.7495062823038834	0.9204618057247105
0.8354877506260885	0.5839535926764904	0.0817394168950703
0.8537067682212262	0.4166666570000004	0.3350336368339107
0.5005022597400568	0.5820320280629868	0.6685491270827090
0.3337279365283514	0.5880872975831867	0.8304446646694393
0.6736991329913675	0.4166666570000004	0.1732827299646209
0.4997921259292349	0.5816353076747910	0.0833775182083126
0.8315339851976534	0.7504847439933314	0.9185676182451348
0.8325232444214136	0.7514235121643112	0.3309285629405024
0.6661091849752491	0.7496420733934757	0.1684920622261497
0.8360114174119465	0.4166666570000004	0.9166850386954501
0.4994958415029859	0.9166666870000029	0.0824054129408996

0.6655677586145934	0.9166666870000029	0.4184048454431563
0.6698287743988263	0.4166666570000004	0.5837109374927979
0.3330222745721897	0.7500733096533783	0.1678527978281267
0.4997719931992277	0.7474484948967234	0.3319977041538046
0.8332305693672906	0.9166666870000029	0.0811227406593493
0.4964544894931440	0.4166666570000004	0.9208451456058281
0.9980890557253584	0.0809505863665675	0.3705317134024665
0.0002012911048076	0.9166666870000029	0.6288024601569971
0.1684164036036719	0.9166666870000029	0.8722119314520422
0.1684914936607611	0.0824959145108176	0.1252702884440973
0.9981119716957899	0.2387265458685902	0.6311022698983249
0.3321172030702528	0.0871765116034388	0.3767270646434602
0.1680237695464987	0.2430444490863514	0.8704359948374059
0.3342758304446372	0.2520646477671593	0.6209494728259534
0.9981119716957899	0.5946067671314073	0.6311022698983249
0.1680237845464964	0.5902888639136457	0.8704359948374059
0.1774195378054110	0.4166666570000004	0.0975324759238859
0.6661332855808629	0.2506504300803226	0.6250073209630627
0.6647252405452838	0.0834470172088612	0.3759296731354494
0.4993180315946646	0.0838471448954744	0.1250329419823139
0.8300701063432583	0.2483878723613393	0.8761412618454647
0.4976301874331798	0.2514687811358124	0.8758251504404493
0.8314561954009201	0.0829939249404446	0.1251289230402001
0.1684914936607611	0.7508374214891850	0.1252702884440973
0.9980890557253584	0.7523827496334349	0.3705317134024665

0.6661260346002819	0.9166666870000029	0.6248847988528985
0.8309886238040095	0.9166666870000029	0.8745484531482712
0.3321172030702528	0.7461568243965635	0.3767270646434602
0.4978399152856458	0.9166666870000029	0.8760625421697315
0.3352861250938630	0.9166666870000029	0.6199996869459043
0.3342758304446372	0.5812686652328379	0.6209494728259534
0.6661332855808629	0.5826828829196742	0.6250073209630627
0.8300701063432583	0.5849454406386581	0.8761412618454647
0.5079299816368235	0.4166666570000004	0.1317156206193060
0.6647252405452838	0.7498863187911412	0.3759296731354494
0.4993180315946646	0.7494861911045284	0.1250329419823139
0.8548950273035991	0.4166666570000004	0.1165762105855372
0.4976301874331798	0.5818645318641916	0.8758251504404493
0.8314561954009201	0.7503394110595584	0.1251289230402001
0.6610003997710918	0.4166666570000004	0.3737492603845780
